# Prevalence and associated factors of minimally invasive facial cosmetic surgery in Chinese college students

**DOI:** 10.1186/s12888-021-03676-3

**Published:** 2022-01-10

**Authors:** Xingyue Jin, Mireille Twayigira, Wenjing Zhang, Xueping Gao, Xuerong Luo, Huiming Xu, Chunxiang Huang, Yanmei Shen

**Affiliations:** 1 National Clinical Research Center for Mental Disorders, and Department of Psychiatry, The Second Xiangya Hospital of Central South University, Changsha, 410011 Hunan China; 2 Changsha University of Science and Technology, Changsha, Hunan 410011 China

**Keywords:** Minimally invasive facial cosmetic surgery, Chinese college students, Risk factors, Prevalence, depression, anxiety

## Abstract

**Background:**

Minimally invasive facial cosmetic surgery (MIFCS) is becoming more and more popular and acceptable in Chinese young people, and it influences people in many aspects. However, there is little research on the associations between MIFCS and psychopathology in Chinese college students. The purpose of this study was to identify the prevalence of MIFCS and its associated factors among Chinese college students.

**Methods:**

A cross-sectional design was applied in this study. A total of 8089 students completed an online questionnaire on demographic data, depressive symptoms (Self-Rating Depression Scale), anxiety symptoms (Self-Rating Anxiety Scale) and MIFCS. Logistic regression was used to identify independent factors associated with MIFCS.

**Results:**

The prevalence of MIFCS in Chinese college students was 2.7% (221/8098). Students with MIFCS were more likely to be from urban areas, from a single child household, experience depression or anxiety and have a history of smoking (all *p* < 0.05). They were also less likely to be right-handed or have a good relationship with father or mother (all p < 0.05). Binary logistic regression showed that older age (OR,1.162; 95%CI [1.061,1.273]), female sex (OR,1.837; 95%CI [1.352, 2.497]), community (urban) (OR,0.601; 95%CI [0.441,0.818]), right-handedness (OR,0.668; 95%CI [0.454,0.985]), depressive symptoms (OR, 4.708; 95%CI [1.690,13.112]), family income (30,000–70,000 yuan per year) (OR,0.572; 95%CI [0.403,0.812]) and smoking (OR,1.571; 95%CI [1.09,2.423]) were independently associated with MIFCS.

**Conclusions:**

Minimally invasive facial cosmetic surgery (MIFCS) is very common in Chinese college students, indicating the importance of paying attention to MIFCS. This study provides valuable evidence for college counselors and doctors in the cosmetic department to provide better and healthier services to students who undergo MIFCS, especially those with depressive symptoms.

## Introduction

Cosmetic surgery, plastic surgery or esthetic surgery, refers to restoring, reconstructing, or improving one’s defective structures using medical techniques [1, 2], and it is often for the face. For general people without deformity, minimally invasive facial cosmetic surgeries (MIFCS) [3] are more acceptable because they are less invasive and less traumatic, they have less postoperative complications and they ensure a quick recovery [4]. These include operative treatments such as double-fold eyelid operation and non-operative treatments, such as botulinum toxin injection or soft tissue filler injection [5]. The primary purpose of cosmetic treatments is to help individuals who are often “normal” feel more attractive and satisfied with their outlooks by creating and maintaining a harmonious look [6, 7].

Cosmetic medicine is becoming more and more popular and acceptable in China, especially in young people. According to the 2019 report on Medical and Beauty Industry published by SoYoung, the biggest medical beauty information platform in China, from 2017 to 2018, the number of cosmetic medical treatment courses increased by 26.4%, exceeding the number in America, Brazil, Japan and Korea, becoming number one globally [8], and the mean age of medical beauty consumers was 24.45 years old [8]. According to the International Society of Aesthetic Plastic Surgery (ISAPS), the entire nonsurgical procedures increased by 11.7%, and total procedures increased by 5.1% in 2018 worldwide [9]. The American Society of Plastic Surgeons (ASPS) showed that there were 18.1 million total cosmetic procedures in the United States in 2019, and 16.3 million of them were minimally-invasive procedures [10]. In previous studies, the prevalence of MIFCS was 6.0% (28/466) [11] in Chinese high school student, and between 9.4% [1] and 9.8% [12] in Chinese college students, while many more students planned to get MIFCS) [1, 11, 12]. Ahmad et al. found that 5.6% of college students in Pakistan have had plastic surgery [13], and in another study in Singapore, the prevalence of cosmetic procedures was 2.5% in junior college students and 3.0% in medical students [14]. Similarly, the number of students who wanted to undergo cosmetic procedures was much higher than the number of students who had already undergone cosmetic procedures [13, 14],which indicates that cosmetic procedures are becoming more and more popular, especially among young people.

Waldman et al. have summarized cosmetic motivations and divided them into eight general categories: (1) mental and emotional health, (2) cosmetic appearance, (3) physical health, (4) work and/or school success, (5) social well-being, (6) cost and/or convenience, (7) procedural perceptions, and (8) timing of treatment [15]. These can be further divided into three aspects: easy access to cosmetic procedures, inner desire to be beautiful, and influences of external perception. Presently, cosmetic treatments have grown more mature compared with the traditional cosmetic operations, especially with the emergence of MIFCS such as hyaluronic acid (HA), botulinum toxin and laser treatments where people suffer less pain [16]. With the fast development of the Internet, people can easily get access to information about esthetic treatments, and with a better understanding, they are more willing to try this beauty operation [17]. From the consumers’ perspective, there are numerous benefits of MIFCS. To be specific, previous studies found that cosmetic procedures improved participants’ quality of life [6, 18–22] and body image [19–22]. For self-esteem however, results have been varying. For example, a systematic review found that minimally invasive facial cosmetic procedures improved participants’ self-esteem [20], and similarly, Dayan et al. found that the injection of botulinum neurotoxin type A (BoNTA) increased participants’ overall self-esteem, appearance-, social-, and performance-related self-esteem [19]. However, Sobanko et al. found that self-esteem was unchanged after minimally invasive cosmetic procedures [21]. Interestingly, some results have shown that compared with those who did not receive esthetic treatments, participants who received esthetic treatments showed a higher quality of life, especially health-related quality of life [23]. Body image and self-esteem have also been associated with motivational factors for cosmetic surgery [22, 24]. Besides these benefits, cosmetic procedures may also lead to complications, such as infections [25], looking worse or even death [26].

In terms of social aspects, beautiful people can have more conveniences, such as better interpersonal relationships, employment opportunities, or financial compensation, and people are often influenced by this stereotype. For instance, Griffin et al. found in their experiments: “Unattractive women are at a disadvantage relative to either medium or attractive women, and it is more often the case that unattractiveness is “bad” while beauty is “good.” [27]. Another study found that students who were less satisfied with themselves and thought appearance was critical were more likely to undergo plastic procedures [1, 12]. People may also be influenced by others to undergo cosmetic procedures. For example, 48.5% of the female college students in Saudi Arabia said that they were influenced by social media or family members or friends when considering undergoing plastic procedure or not [28].

Previous studies have revealed associations between MIFCS and participants’ mental health [29–31]. Additionally, given that college students are more likely to come across and try new things which makes them a susceptible population for a new thing such as MIFCS, there have been several studies in foreign countries investigating the prevalence of cosmetic procedures among college students [13, 14]. However, the sample sizes of these studies have not been large enough [1, 11, 12], and in addition, little research has been done among Chinese college students. Therefore, the purpose of this study was to investigate the prevalence of MIFCS and the associated psychological factors. We hypothesized that college students with MIFCS would show higher levels of anxiety and depressive symptoms. As far as we know, our study is the first of its kind to investigate the prevalence of MIFCS and its relationship with psychological health among Chinese college students in a large sample size.

## Material and methods

### Participants

This study was approved by the ethics committee of the Second Xiangya Hospital. A cross-sectional design was used in this study between February 2019 and June 2019 with convenience sampling method. We designed a set of questionnaires geared to college students. With college counselors’ help, we distributed the questionnaires online at Changsha Medical University and Changsha University of Science and Technology through WeChat, a commonly used social media platform in China. Each college counselor was trained to deliver and guide students to fill in the questionnaires. A total of 8130 students were invited to participate in the survey, 2085 from Changsha Medical University and 6045 from Changsha University of Science and Technology. Each student was informed that what they filled in the questionnaires was only to be used for scientific research and their personal information would not be disclosed in any situation. They could decide whether to participate or not, and they could withdraw at any time if they did not want to continue. The survey was conducted anonymously. All participants provided informed consent to participate in this study.

The inclusion criteria were 1) participants aged 17 to 25 years old; 2) Those in good health; and 3) Those who agreed to participate in this study and signed electronic informed consent. Students with severe physical illness were excluded from the study. Finally, five students refused to participate, 27 students were excluded because they did not complete all questionnaires, so the data of 8089 students were taken for further analysis.

### Measures

We collected demographic data of each student, including their sex, age, right-handedness, nationality, community (urban or rural), one-child family, parents’ education level, family income, a good relationship with parents, history of physical disorders, history of mental disorders, family history of mental disorders, alcohol drinking and smoking habits. Students who drink more than once a week on average, and drink more than 25 ml liquor, 250 ml beer or 50 ml wine were considered as “drinking alcohol” group [32, 33]. Students who never drink alcohol or drink less than the frequency and quantity mentioned above were considered as “no drinking alcohol” group. Students who never smoke or smoke less than one cigarette a day, less than five cigarettes a week on average were considered as “no smoking” group. Students who smoke more than one cigarette a day and at least five cigarettes a week were considered as “smoking” group [34].

We also collect information on their psychological health, including depressive symptoms and anxiety symptoms. Depressive symptoms were measured using the Self-rating Depression Scale (SDS) [35]; a total standardized score that reached or exceeded 53 was considered as having depressive symptoms. Anxiety symptoms were measured using the Self-rating Anxiety Scale (SAS) [36]; a total standardized score that was equal to or higher than 50 was considered as having anxiety symptoms.

After explaining the definition of Minimally Invasive Facial Cosmetic surgery (MIFCS), questions about MIFCS were asked using the following questions: “have you ever undergone any MIFCS.” For students who answered “No”, further questions were asked on why they had not. For students who answered “Yes”, further questions on what kind of MIFCS, why they underwent MIFCS and whether MIFCS lived up to their expectations were asked.

### Statistical analysis

In this study, Kolmogorov–Smirnov one-sample test was utilized to exam whether the continuous data met the Gaussian distribution. The data of age met the Gaussian distribution, and student t-test was employed to compare the average age of students with and without MIFCS. For categorical variables such as sex, community, nationality, etc., Chi-square test was used to compare the group differences. Binary logistic regression was utilized to calculate the adjusted OR for MIFCS, and method was set as enter, whether or not have MIFCS were put as the dependent variable, all the other variables in Table 1 were set as covariates to control for the confounding variables, which was used to determine which variables were independently associated with MIFCS and calculated their adjusted OR. All statistical analyses were completed using SPSS (Version 22.0; IBM, Inc., Chicago, IL), the statistical significance level was set at *p* < 0.05(two-tailed).

**Table 1:** Demographics of participants with and without minimally invasive facial cosmetic surgery (MIFCS)

Variables	Students with MIFCS *N* = 221		Controls *N* = 7877				
	n	%	n	%	χ^2^/t	df	p
Age,mean ± SD	20.52	1.52	20.23	1.50	8.257	1	.004
Sex							
Male	81	36.70	3511	44.60	5.465	1	.019
Female	140	63.30	4366	55.40			
Community							
Urban	124	56.10	3318	42.10	17.206	1	<.0001
Rural	97	43.90	4559	57.90			
Nationality							
Han	199	90.00	7159	90.90	0.183	1	.669
Others	22	10.00	718	9.10			
Physical disorder history	12	5.40	247	3.10	3.654	1	.056
Mental disorder history	4	1.80	108	1.40	0.304	1	.582
Family history of mental disorder	5	2.30	103	1.30	1.489	1	.222
Right handedness	186	84.20	7087	90.00	7.925	1	.005
Good relationship with mother	203	91.90	7598	96.50	12.891	1	< 0.0001
Good relationship with father	200	90.50	7470	94.80	8.071	1	.004
Single child household	105	47.50	3135	39.80	5.327	1	.021
SDS, mean ± SD	50.87	11.93	44.35	9.89	94.396	1	<.0001
Depressive symptoms (yes)	61	27.60	956	12.10	46.82	1	<.0001
SAS, mean ± SD	45.38	11.71	39.91	9.35	75.39	1	<.0001
Anxiety symptoms (yes)	65	29.40	1265	16.10	27.921	1	<.0001
Family income							
< 30,000 yuan	68	30.80	1964	24.90	12.528	2	.002
30,000–70,000 yuan	65	29.40	3248	41.20			
> 70,000 yuan	88	39.80	2665	33.80			
Smoking	31	14.00	700	8.90	6.917	1	.009
Drinking	83	37.60	2500	31.70	3.35	1	.067

*Note*: Minimally Invasive Facial Cosmetic surgery = MIFCS, standard deviation = SD, Self-rating Depression Scale = SDS, Self-rating Anxiety Scale = SAS

all the variables were put into the binary logistic regression model to control for the confounding variables.

## Results

The mean age in this sample was 20.24 (SD = 1.50), with 44.4% male and 55.6% female. The prevalence of MIFCS among Chinese college students was 2.7% (221/8098), and the prevalence in female students was significantly higher than that in male students (63.30% vs. 36.70%, *p* = 0.019). Additionally, students with MIFCS were more likely to be from urban areas, from a single child household, to have depressive or anxiety symptoms and to have a history of smoking (all *p* < 0.05); they were less likely to be right-handed and to have a good relationship with father or mother (all p < 0.05). No significant differences were observed between students with and without MIFCS in nationality, physical disorder history, mental disorder history, family history of mental disorder and drinking alcohol. (Table 1).

Additionally, the percentages of students in different categories of MIFCS were also calculated; 97.3% of the participants received no surgery, 1.6% underwent one kind of surgery, 1.2% received more than one kind of surgery.

**Fig. 1:**
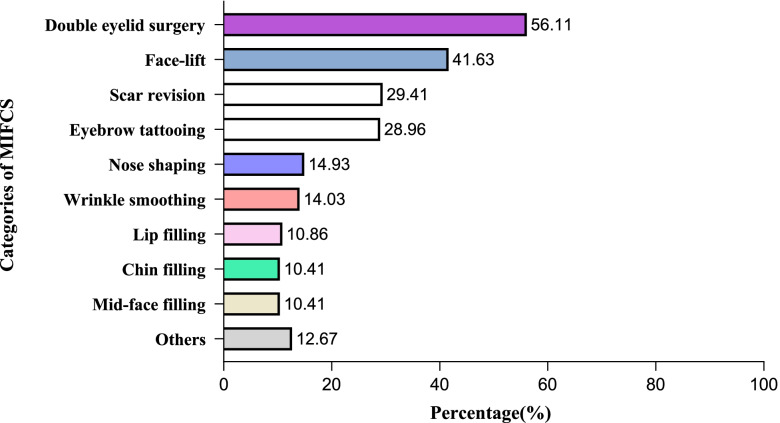
The percentage of each category of Minimally Invasive Facial Cosmetic surgery (MIFCS) college students underwent. Note: Minimally Invasive Facial Cosmetic surgery = MIFCS. The length of rectangles represents the percentage of students who underwent each type of MIFCS

Considering the types of MIFCS, double eyelid surgery (124, 56.11%) and face-lift (92, 41.63%) were more common in students with MIFCS (Table 2, Fig. 1). The main reasons for MIFCS were dissatisfaction with one’s own looks (35.70%) or boosting confidence (28.50%). Among the students who had not undergone MIFCS, the main reason for not having these facial surgeries was that they thought it was unnecessary because the original beauty was enough. Other reasons for not having MIFCS included economic conditions that could not allow it (10.70%), satisfaction with one’s own looks (12.60%) and safety concerns about MIFCS (10.00%). Most of the students who received MIFCS were satisfied with their cosmetic procedures, as high as 81.40% of them thought the plastic surgeries lived up to their expectation. (Table 2).

**Table 2:** Description of minimally invasive facial cosmetic surgery (MIFCS) related constituent ratio

Variables	Students with MIFCS		Controls	
	n	%	n	%
Categories of MIFCS				
Face-lift	92	41.63		
Double eyelid surgery	124	56.11		
Scar revision	65	29.41		
Eyebrow tattooing	64	28.96		
Mid-face filling	23	10.41		
Chin filling	23	10.41		
Nose shaping	33	14.93		
Lip filling	24	10.86		
Wrinkle smoothing	31	14.03		
Others	28	12.67		
Reasons for undergoing MIFCS				
Unsatisfied with appearance	79	35.70		
For work	15	6.80		
For finding a mate	11	5.00		
For Boosting confidence	63	28.50		
Others	53	24.00		
Effectiveness of MIFCS				
Expectations met	180	81.40		
Expectations not met	41	18.60		
Reasons for not undergoing MIFCS				
Economic pressure			840	10.70
Satisfied with present self			996	12.60
Not necessary			5258	66.70
Not safe enough			784	10.00

*Note*: Minimally Invasive Facial Cosmetic surgery = MIFCS

Binary logistic regression analysis was conducted to identify factors independently associated with MIFCS, and the reference group of categorical variables in binary logistic analysis were as follows: Sex (male), community (Urban), nationality (Han), physical disorder history (No), mental disorder history (No), family history of mental disorder (No), right handedness (No), good relationship with mother (No), good relationship with father (No), single child hold (No), depressive symptoms (No), anxiety symptoms (No), family income (< 30,000 yuan), smoking (No), drinking alcohol (No). The results showed that female students were more likely to accept MIFCS (OR = 1.837, 95%CI [1.352, 2.497], *p* < 0.001). Students from rural areas were less likely to undergo MIFCS (OR = 0.601, 95%CI [0.441, 0.818], *p* = 0.001). Students with depression symptoms (OR = 4.708, 95%CI [1.690, 13.112]) and smoking history (OR = 1.571, 95%CI [1.019, 2.423], *p* = 0.041) were more likely to undergo MIFCS. Additionally, compared with a family income less than 30,000 yuan/year, students with a family income between 30,000 to 70,000 yuan/year showed less probability of having MIFCS (OR = 0.572, 95%CI [0.403, 0.812], *P* = 0.002); however, the possibility of getting MIFCS was not significantly different between family income less than 30,000 yuan and more than 70,000 yuan (OR = 0.879, 95%CI [0.625, 1.236], *p* = 0.459). (see Table 3).

**Table 3:** Logistic regression analysis for variables associated with minimally invasive facial cosmetic surgery (MIFCS)

Variables	B	S.E.	Wald	df	*p* value	OR	95% CI
Age	.150	.046	10.554	1	.001	1.162	1.061–1.273
Sex							
Male	reference						
Female	.608	.157	15.086	1	< 0.001	1.837	1.352–2.497
Community							
Urban	reference						
Rural	−.510	.157	10.484	1	.001	.601	0.441–0.818
Nationality							
Han	reference						
Others	.012	.231	.003	1	.957	1.013	0.644–1.592
Physical disorder history							
No	reference						
Yes	.317	.350	.820	1	.365	1.373	0.692–2.724
Mental disorder history							
No	reference						
Yes	−.865	.713	1.475	1	.225	.421	0.104–1.701
Family history of mental disorder							
No	reference						
Yes	.436	.627	.483	1	.487	1.546	0.452–5.282
Right handedness							
No	reference						
Yes	−.403	.198	4.150	1	.042	.668	0.454–0.985
Good relationship with mother							
No	reference						
Yes	−.578	.352	2.692	1	.101	.561	0.281–1.119
Good relationship with father							
No	reference						
Yes	−.053	.329	.025	1	.873	.949	0.498–1.809
Single child hold							
No	reference						
Yes	.172	.154	1.253	1	.263	1.188	0.878–1.607
Depressive symptoms							
No	reference						
Yes	1.549	.523	8.788	1	.003	4.708	1.690–13.112
Anxiety symptoms							
No	reference						
Yes	−.657	.512	1.649	1	.199	.518	0.190–1.413
Family income			10.959	2	.004		
< 30,000 yuan	reference						
30,000–70,000 yuan	−.558	.179	9.758	1	.002	.572	0.403–0.812
> 70,000 yuan	−.129	.174	.549	1	.459	.879	0.625–1.236
Smoking							
No	reference						
Yes	.452	.221	4.180	1	.041	1.571	1.019–2.423
Drinking alcohol							
No	reference						
Yes	.199	.157	1.612	1	.204	1.220	0.898–1.658

Note: *CI* confidence interval, *OR* odds ratios

## Discussion

This study was designed to investigate the prevalence of minimally invasive facial cosmetic surgery (MIFCS) and its related psychological risk factors among a large sample of college students in Hunan province, China. We investigated 8098 college students and found that the prevalence of MIFCS in college students was 2.7%. Students with and without MIFCS had differences in the aspects of sex, family relationship and psychopathology. Moreover, after controlling for confounders, age, sex, community, right-handedness, depressive symptoms, family income and smoking were independently associated with MIFCS.

We found that the overall prevalence of MIFCS in college students in Hunan province was 2.7%, which was much lower than the prevalence reported in other cities of China such as Guangzhou (9.4%) [1], Inner Mongolia (9.8%) [12], and Yangzhou (6.0%) [11]. The discrepancy in prevalence might be explained by several reasons. Firstly, the definitions of MIFCS in each study was different. In Hu’s study, circumcision and tooth flattening were included as MIFCS; however, these two were not included as MIFCS in this study [11]. The differences in sex ratio may be another factor. In Yang’s study, the percentage of females was a bit higher than ours (60.3% vs. 55.64%) [1]. Secondly, our study was conducted in a much larger sample, so the results are relatively more representative and less influenced by sampling methods bias. Compared with foreign studies, the prevalence of MIFCS of this study was similar to Singapore (2.5% for junior college students and 3.0% for medical students) [14], but lower than the prevalence in Pakistan (5.6%) [13] and that shown by American facial plastic surgeons (75%) [37]. The differences may be due to the accessibility of MIFCS, as well as values and attitudes towards MIFCS. The International Society of Aesthetic Plastic Surgery (ISAPS) showed that the United States has the most surgeons and most nonsurgical procedures [9]; meanwhile, plastic technology is much more mature in foreign countries such as Korea, therefore many Chinese patients decide to visit Korea for plastic surgery or choose plastic surgery done by Korean doctors [38].. Thirdly, economic reasons might also play part. Paik et al. revealed that the total number of plastic surgery procedures was significantly positively related to Gross Domestic Product (GDP), GDP per capita and personal income [39], which can partly explain the rapid increase of cosmetic procedures in China from 2017 to 2018 [8]. Finally, culture might also contribute to this lower prevalence, for there is an old Chinese saying that says “My skin and hair are given by my parents and cannot get any scratches” [40].. Moreover, Chinese people have been more conservative and advocate for the natural beauty, therefore the attitude towards plastic procedures has not been positive in past years and the prevalence of MIFCS has been much lower.

This study also found that the prevalence of MIFCS in females was higher than that in male students (3.11% vs. 2.26%, *p* = 0.019). Although SoYoung platform showed that nearly 90% of the customers who underwent MIFCS were female, the number of males undergoing MIFCS increased by 52.3% from 2017 to 2018 [8]. In the United States, the percentage of cosmetic procedures in male patients has increased by 273% from 1997 to 2015 [41]. In America, the entire cosmetic procedures in males increased by 27% and the total cosmetic minimally-invasive procedures in males increased by 72% in 2019 compared with those in 2000 [10]. This indicates that although females are the main consumers of MIFCS, the number of males is also on the rise. This might be due to the fact that appearance has a certain influence on one’s daily life. Zeng et al. found that good appearance had a significant positive effect on one’s income [42]. Another study suggested that appearance plays an important role in marital satisfaction [43], and people may also demand more of women’s appearance. When choosing a mate, males pay more attention to the appearance of their potential partners [44]. Additionally, females have a greater internalization of mass media images of beauty [28]. On the other hand, males seem to also be pushed into the direction of “Appearance is Power”. A study in Thailand showed that peer pressure influenced males to seek cosmetic services because appearance influenced how they are perceived in their workplace, their self-esteem and confidence [45]. With the propaganda of all kinds of media, people are deeply influenced by osmosis. Every day, the people we see on televisions, laptops, smartphones or even commodities are all similarly beautiful, but maybe not so realistic. The men seen in the public eye probably have deep double eyelids, high nose bridge, attractive lips, fair skin, an angular face, which looks as pretty as a woman’s face. In this case, people’s esthetic standards are affected inevitably, and in order to meet these standards, the desire for MIFCS or plastic surgery increases spontaneously.

This study also found that college students who had undergone MIFCS had a significantly higher level of depressive symptoms and anxiety symptoms. After controlling for confounding factors, we found that depressive symptoms were independently associated with MIFCS. One previous study has found that 22.3% (223/1000) of the patients seeking elective plastic surgery in America suffered from major depressive disorder, and 14.5%(145/1000) of them experienced generalized anxiety disorder [29]. Soest et al. found that symptoms of depression and anxiety could predict prospective surgery (OR = 1.66, 95%CI [1.07–2.57]) and those who underwent surgery experienced a greater increase in the symptoms of depression and anxiety [30]. Interestingly, another study showed that plastic surgery may also reduce participants’ anxiety and depression level. Moss et al. found that people who underwent plastic surgery experienced less symptoms of depression and anxiety post-operatively [31]. These relationships may be due to the following reasons: Valikhani et al. pointed out the influences of “self-knowledge”, they thought people with high self-knowledge know their “selves”, hence they rely more on themselves, accept themselves and consider a non-conditional worth for themselves, instead of attracting the attention of others by such things as appearance [46]. People who seek plastic surgery or MIFCS may have low self-knowledge [46], more neurotic [23] and more sensitive to negative experiences. They may think that they are unable to attract others’ approval by their appearance, so they may unconsciously suffer from negative feelings such as depression or anxiety. People being teased because of appearance are more likely to be interested in cosmetic surgery [47]. In the Internet era, most of the portraits we see online are comely and fascinating, such as in films and television, or in social media like Weibo, WeChat, Facebook and Twitter. However, social media is also associated with increased anxiety and feelings of inadequacy [48]. Looking better in photos is a primary motivator for seeking plastic surgery [49]. When photos are posted on social media, beautiful photos can receive more compliments, otherwise feelings of depression and anxiety may be triggered. However, Others hold a different view, that people who undergo minimally invasive facial rejuvenation procedures have more positive and protective personality traits, that they are more open to experiences, more extroverted, more agreeable [23].

Several limitations need to be noticed in this study. Firstly, it is a cross-section design study, so even though this study revealed the association between MIFCS and psychological factors, we could not draw any causality conclusions. A longitudinal study is needed to further explore the causal relationship between these variables. Secondly, some other important variables were not measured in this study, more features such as body dysmorphic disorder need to further explored in future research. Thirdly, although our study was done using a large sample size, all data was collected in colleges of the same city; therefore, caution should be taken when extending the conclusions to larger areas. Fourthly, the survey was anonymous, and although we protected the privacy of participants, we could not offer more help to those who had adverse mental health symptoms. Fifthly, this study conducted a spearman correlation analysis to explore the relationship between these variables, further in-depth analysis is warranted in future studies.

## Conclusion

In conclusion, the prevalence of minimally invasive facial cosmetic surgery (MIFCS) among Chinese college students in Hunan province was 2.7%. Age, sex, community, right-handedness, depression, family income and smoking were independently associated with MIFCS. This study provides valuable evidence for college counselors and doctors in the cosmetic department to take care of the students who undergo MIFCS. It also emphasizes the importance of education aiming to establish a proper aesthetic view in college students.

## Data Availability

The data that support the findings of this study are available from the corresponding author upon reasonable request.
